# Infrared spectroscopy with statistical analysis and machine learning for cancer risk assessment in inflammatory bowel disease patients

**DOI:** 10.1039/d6an00649c

**Published:** 2026-09-14

**Authors:** Eric Kumi-Barimah, Raneem Toman, Animesh Jha, Sharib Ali, Venkataraman Subramanian

**Affiliations:** a School of Chemical and Process Engineering, University of Leeds Leeds LS2 9JT UK E.Kumi-Barimah@leeds.ac.uk; b Southwest Jiatong University-University of Leeds Joint School Chengdu 610031 China; c School of Computer Science, University of Leeds Leeds LS2 9JT UK; d Leeds Institute of Medical Research at St James's, St James's University Hospital, University of Leeds Leeds LS9 7TF UK

## Abstract

Patients with inflammatory bowel disease (IBD) undergo regular colonoscopic surveillance due to their elevated risk of developing colorectal cancer (CRC). However, current clinical, endoscopic and histopathological risk stratification methods can be limited in sensitivity and objectivity, highlighting the need for complementary molecular approaches. In this study, Attenuated Total Reflectance–Fourier Transform Infrared Spectroscopy (ATR-FTIR) was used to acquire mid-infrared (MIR) spectra from IBD-associated endoscopic biopsy samples (30 patients analysed; 10 who developed dysplastic pre-cancerous lesions and 20 who did not during long-term follow-up). Samples were stratified according to baseline clinical CRC risk (high/low) and subsequent pre-cancerous lesion development, enabling assessment of molecular signatures associated with future cancer risk rather than solely current disease status. Spectral variations were analysed using chemometric methods and machine learning classifiers. Principal component analysis (PCA) was performed to evaluate spectral separation, with the first three components (PC1, PC2 and PC3) accounting for 76.8%, 19.2% and 2.7% of the total variance, respectively. Hierarchical clustering analysis (HCA) further explored similarity patterns across defined spectral regions. Among the evaluated models, PLS-based classifiers achieved a balanced accuracy of 0.61–0.70 under repeated nested cross-validation; however, a permutation test indicated that this performance was not significantly above chance (*p* = 0.51), and no classifier significantly outperformed another. These findings indicate that ATR-FTIR-derived molecular fingerprints, particularly in the lipid-associated 2950–3030 cm^−1^ region, carry information associated with long-term CRC risk in IBD patients, but that a definitive classifier cannot be established from a cohort of this size; the approach is presented as a complementary, hypothesis-generating tool to conventional surveillance strategies.

## Abstract 2

Patients with inflammatory bowel disease (IBD) undergo regular colonoscopic surveillance due to their elevated risk of developing colorectal cancer (CRC).

## Graphical abstract

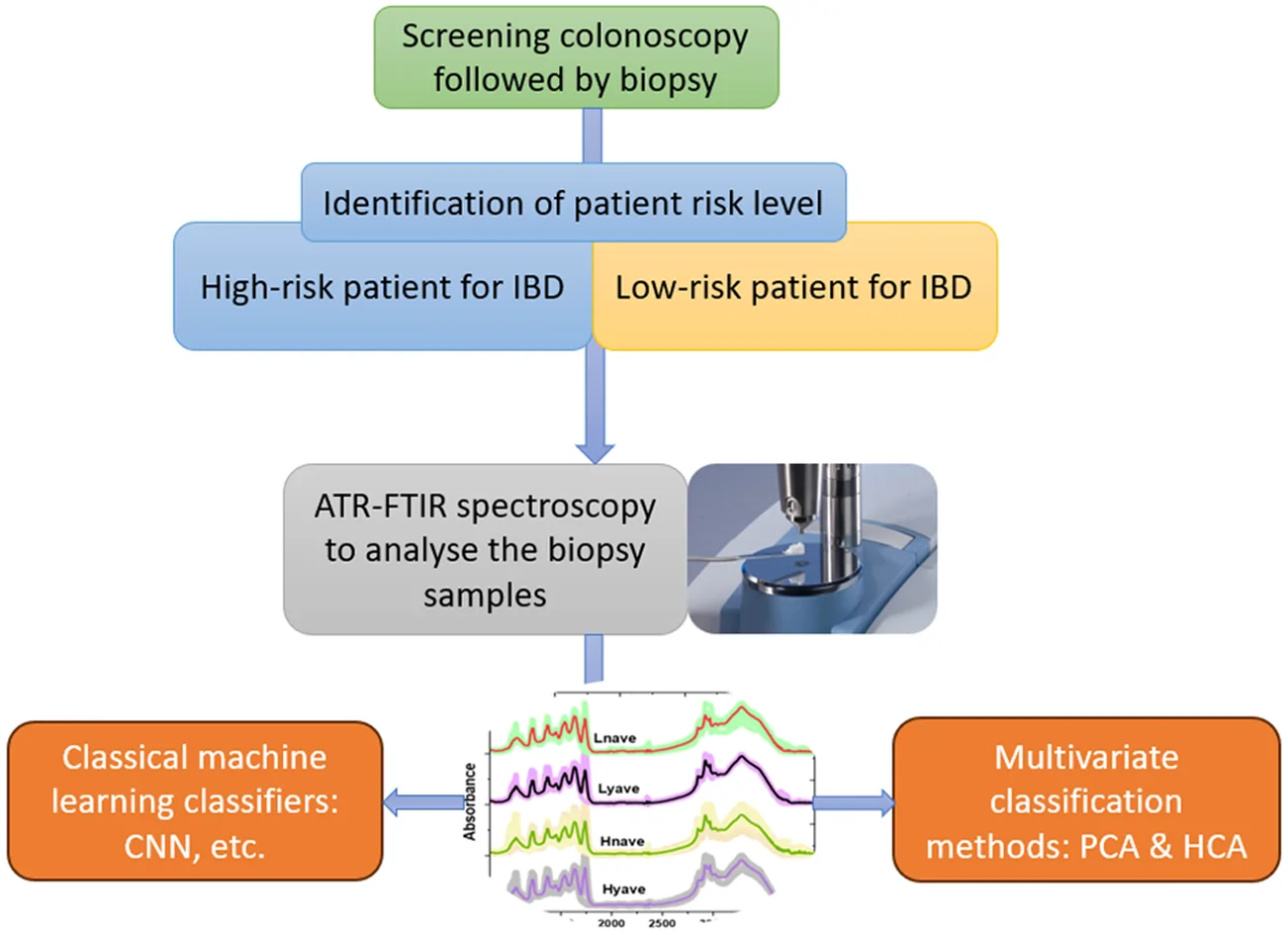

## 1 Introduction

Inflammatory bowel disease (IBD) is a chronic inflammatory disorder of the gastrointestinal tract, primarily comprising Crohn's disease (CD) and ulcerative colitis (UC), both of which affect the colon.^1^ Patients with IBD are at increased risk of developing dysplasia, including polypoid and non-polypoid lesions, which can progress to colorectal cancer (CRC).^1–3^ This increased risk of CRC in colonic IBD has been recognised for nearly a century and has been corroborated by several large population-based studies and meta-analysed over the past two decades.^4,5^ Although recent epidemiological studies report a decline in IBD-related CRC incidence due to advances in surveillance and screening, individuals with IBD still have approximately twice the risk of developing CRC compared to the general population.^6^ According to the Global Cancer Observatory (2022) and the GLOBOCAN database (2023), CRC ranks third in global incidence and second in global mortality.^7,8^ The early diagnosis of IBD-related CRC leads to a higher survival rate of 98% compared to 44% for those diagnosed late.^9^ Current risk stratification relies mainly on clinical history, endoscopic findings and histopathology.^10^ However, these methods can be limited when it comes to achieving high sensitivity and specificity for early CRC diagnosis.

CRC within colonic IBD is believed to progress along an inflammation–dysplasia–carcinoma sequence, although this transition can be accelerated and does not always conform strictly to a stepwise process.^11,12^ Advances in endoscopic imaging have improved the detection of visible dysplasia; however, current imaging approaches do not consistently allow conclusive identification of lesions, particularly when obscured by active inflammation. Colonoscopic surveillance using dye-spray chromoendoscopy remains the gold standard for detecting dysplasia in IBD surveillance programs.^13,14^ Surveillance intervals are determined according to national and international risk stratification guidelines based on clinical and pathological factors. However, the evidence underpinning these stratification systems is neither uniformly robust nor fully generalizable, leading to potential misclassifications of patients.^13^ Consequently, some individuals may undergo unnecessary colonoscopies, whereas others at higher risk may not receive sufficiently intensive surveillance.^6,15^ These limitations may contribute to the observation that post-colonoscopy (interval) CRC rates are approximately two to three times higher in patients with IBD compared with the general population.^16^ Furthermore, more than 10% of missed CRC cases in England and Wales occur in patients with IBD, and clinical outcomes are often worse and occur in younger individuals within this cohort.^17^

The concept of “field cancerization” or the “field effect” provides a biological explanation for some of these diagnostic challenges. Field cancerization refers to the presence of widespread molecular and genetic alterations in histologically normal-appearing mucosa surrounding or distant from a tumour.^18^ In IBD, chronic inflammation can induce diffuse genetic and epigenetic changes across the colonic epithelium, thereby obscuring discrete lesion boundaries. Genetic mutations have been identified in non-dysplastic mucosa remote from tumour sites, including TP53 mutations,^12^ KRAS mutations,^19^ microsatellite instability,^20^ and epigenetic alterations such as aberrant DNA methylation and histone modification.^21,22^ These diffuse molecular alterations can precede morphological change, limiting the sensitivity of purely visual endoscopic assessment and histological sampling.

Colonoscopy with biopsy histopathology surveillance programs are widely recognised as the gold standard for reducing the risk of IBD-related CRCs; the colonoscopy techniques provide information that focuses solely on the morphological characteristics observed along the colon, but fail to identify any biochemical changes that can occur. Conversely, the main significant drawbacks are their invasiveness, potential for complications, the requirement for bowel preparation, resource demands and associated costs.^23^ Besides, the interpretation of the results can be affected by the observer's subjective judgment,^24^ leading to the missed diagnosis of IBD-related CRC. There are several reasons for the higher rates of late-stage colorectal cancer in IBD. Low rates of adherence to current colonoscopy surveillance protocols is a major reason. A large multi-center French study showed that only 54% of eligible patients with IBD attended for a surveillance colonoscopy over a 41 month period.^25^ Colonoscopy is a resource-intensive procedure, and patients often dislike both the procedure and the bowel preparation laxatives, which could explain the low adherence rates. Therefore, we hypothesise that there is a need to develop and optimise surveillance techniques that identify those with higher risk of IBD-associated CRC to reduce the risk of missed lesions. By the early identification of those at risk of progression we can enhance surveillance in high-risk patients and increase surveillance intervals in lower-risk patients safely.

Considering the limitations, current risk stratification methods, and the biological basis of the field effect, vibrational spectroscopic techniques can offer complementary molecular-level assessment. This work focuses on the application of Fourier Transform Infrared (FTIR) vibrational spectroscopic analysis of dysplasia/cancer-specific spectral biosignatures in randomly selected rectal biopsies, which can harbour molecular alterations not visible during colonoscopic imaging due to field cancerization. Over the years, the Attenuated Total Reflectance–Fourier Transform Infrared Spectroscopy (ATR-FTIR) technique has proven to be a valuable tool for detecting tissue biochemical alterations at the molecular level, which can be used to differentiate between IBD-related CRC precursor and normal tissue.^26,27^ ATR-FTIR spectroscopy has already been employed to identify the molecular fingerprints of several biological tissues and shifts in vibration modes.^28^ Particular attention has been focused on analysing cancerous tissues to determine pathological changes in biochemical molecules such as carbohydrates, polysaccharides, proteins, amino acids, lipids, fatty acids and nucleic acids. For example, FTIR and ATR-FTIR spectroscopy have been extensively used for screening and diagnosing various types of cancer, including ovarian, esophageal, lung, breast and CRC, with numerous reports published to date.^29–35^ For example, Li *et al.*^30^ performed real-time and rapid detection of malignant tissue *in vivo* and *in situ* during surgical operations using the ATR-FTIR fibre-optical spectroscopy technique. The mid-infrared spectra showed significant differences between normal and malignant CRC tissues in both *in vivo* and *in situ* measurements. Su *et al.*^29^ provide a comprehensive review of how FTIR and ATR-FTIR methods can be used to analyse various biological samples, including cancer tissues, highlighting the rationale and advantages of these techniques in cancer diagnosis. Li *et al.*^31^ employed a mobile WQF-500 FTIR spectrometer with an ATR probe to analyse spectra from paired carcinoma and nearby normal tissues in colorectal cancer patients. Furthermore, Yang *et al.*^32^ applied ATR-FTIR spectroscopy to study lung cancer tissues and compare them with tissues from healthy individuals. FTIR spectroscopy has also been used to investigate infrared signatures in cancer cells, blood samples, and colorectal cancer. A total of 180 CRC samples were collected from 90 patients.^35^ These samples were then analysed using FTIR. The study focused on analysing spectral and relative intensity ratios to distinguish between malignant and normal colorectal tissue. It utilised advanced statistical techniques, specifically principal component analysis (PCA) and Fisher's discriminant analysis (FDA), to enhance the differentiation between the two tissue types. In addition, 88 endoscopic biopsy samples of colorectal tissue, comprising 41 cases of colitis and 47 cases of cancer, were collected and analysed using ATR-FTIR fibre probe spectral analysis.^36^ The sample spectra were evaluated using the entropy weight local-hyperplane *K*-nearest neighbour (EWHK) tissue classification method. The EWHK classifier demonstrated a sensitivity of 81.4% and a specificity of 92.7% in distinguishing between cancer and colitis samples, based on the average results of the random classification. Mid-infrared (MIR) FTIR spectroscopy shows promise for screening and diagnosing various human cancers, particularly CRC.

Several chemometric techniques have been developed to correctly predict the differentiating models between healthy and CRC tissues.^37^ PCA and Hierarchical Clustering Analysis (HCA) are widely recognised unsupervised learning techniques. They have been effectively employed to create models that quantitatively predict spectroscopic data. Conversely, Partial Least Squares Regression (PLS-R) is a supervised statistical method for reducing the number of correlated variables in a dataset by creating new ones. Therefore, combining these modelling techniques can help avoid reaching statistically insignificant conclusions and improve our understanding of the classification algorithm for the stratification of CRC risk in patients with colonic IBD.^38–40^ Apart from conventional chemometric analysis, machine learning (ML) classifiers and deep learning (DL) have been successfully applied for diagnosing various types of cancer.^41–45^ The use of convolutional neural networks (CNNs) has demonstrated strong performance in analysing malignant sites within medical images and FTIR spectral signals for cancer diagnosis.^42,46^

In this study, we performed ATR-FTIR spectroscopy analysis on frozen IBD rectal biopsy samples collected during IBD surveillance colonoscopy. The mid-infrared (MIR) absorbance spectra of the IBD-associated CRC samples, spanning the range of 900 to 4000 cm^−1^, were acquired and analysed. The resulting spectral data were evaluated using conventional chemometric multivariate methods, including peak intensity ratios, principal component analysis (PCA) and hierarchical clustering analysis (HCA), to classify risk-stratified patient groups. Moreover, we assessed the performance of different machine learning (ML) classifiers for the detection of cancerous tissue within the biopsies. Overall, this study aims to further validate the use of ATR-FTIR spectroscopy, combined with chemometric and ML-based approaches, to accurately predict the likelihood of CRC in IBD patients compared to benign conditions. The proposed methods seek to enable effective risk stratification of IBD-associated CRC independently of tissue morphological alterations, thereby complementing conventional surveillance strategies and clinical risk prediction tools.

## 2 Materials and methods

### 2.1 Sample preparation

This study received ethical approval from the Leeds Teaching Hospital (LTHT) NHS Trust and the Yorkshire and Humber–Yorkshire Bridge National Research Ethics Committee (reference number 17/EM/0033). Written informed consent was obtained from all participating patients. The samples were collected as part of a parallel-group randomised controlled trial (ClinicalTrials.gov identifier: NCT03250780), in which patients undergoing surveillance endoscopy for colonic inflammatory bowel disease were randomised to either high-definition-chromoendoscopy (HDCE) using 0.2% indigo carmine (IC) applied through a spray catheter or HDCE using 0.03% IC delivered *via* a foot pump. Two random rectal biopsies were snap-frozen in liquid nitrogen and subsequently stored at −80 °C from patients who provided consent for additional research biopsies.

In this study, 34 patients provided consent for additional biopsies to be snap frozen for spectroscopic studies. A total of 60 colonic biopsy samples of dimensions range from 2 mm × 1.5 mm × 1 mm to 2 mm × 2 mm × 1.5 mm were collected during colonoscopy, snap-frozen in liquid nitrogen, and stored at −80 °C. Following removal of one duplicate record and exclusion of three samples that did not meet spectral quality-control criteria, 30 patients were retained for analysis (Table 1). The patients were clinically divided into two categories based on the 2010 guidelines from the British Society of Gastroenterology (BSG): high-risk and low-risk groups. These patients were followed up for at least 6 years (until June 2025) after their surveillance colonoscopy to check whether there was evidence of dysplastic (precancerous) polyps. During follow-up, 5 patients in the high-risk group and 5 patients in the low-risk group developed dysplastic polyps, as shown in Table 1.

**Table 1:** Distribution of patients according to baseline risk level and subsequent dysplasia development

Cancer risk level	Developed dysplasia	Number of cases
High (H)	Yes (Hy)	4
High (H)	No (Hn)	10
Low (L)	Yes (Ly)	5
Low (L)	No (Ln)	15

### 2.2 ATR-FTIR spectroscopy

MIR absorbance spectra of healthy and cancerous colorectal tissue were measured using a Bruker Vertex 70v FTIR spectrometer with an ATR accessory. Frozen samples were allowed to thaw at room temperature for 2–3 minutes before measurement. A background spectrum was first recorded, and the thawed sample was mounted at a 90-degree angle on the diamond crystal of the ATR. Spectra were collected over the wavenumber range 900–4000 cm^−1^, with 4 cm^−1^ resolution and an average of 32 scans to improve the signal-to-noise ratio. A sampling area of 600 μm × 600 μm was analysed, and three spectra were recorded per sample and averaged for accuracy.

### 2.3 Quality control procedures

To ensure data reliability and consistency, we applied various quality control measures during data collection and analysis. We prepared all samples under standardised biopsy conditions. The samples were allowed to dry for 2–3 minutes to minimise water interference. We verified instrument quality through background scans, wavelength calibration using standards such as polystyrene film, and monitoring the signal-to-noise ratio. Acquired spectra were assessed for baseline stability, absence of saturation, low noise, and reproducibility; outlier spectra were excluded, and the measurement was repeated before data analysis. Consistent sample preparation and spectral preprocessing are essential to obtain reliable, reproducible ATR-FTIR results.

### 2.4 Spectral processing and multivariate analysis

Spectral collection and processing were performed using OPUS 5 software. Straight-line subtraction and baseline correction were applied using the rubber-band method, followed by Savitzky–Golay smoothing (17 points), vector normalisation and scattering correction. Multivariate classification methods, including principal component analysis (PCA) and hierarchical clustering analysis (HCA), were applied to differentiate the four IBD-related colorectal categories using OriginPro (OriginLab Corp., USA). PCA was performed on the 900–4000 cm^−1^ range using three components to distinguish between Hy, Ly, Hn and Ln categories. HCA was conducted to determine inter-spectral distances and cluster the spectra, focusing on the 900–1800 cm^−1^ and 2800–3000 cm^−1^ regions.

### 2.5 Classifier selection

We evaluated a range of classical machine learning classifiers together with a one-dimensional convolutional neural network (1-D CNN) tailored to small-sample biomedical data. The classical classifiers included support vector machines (SVM), logistic regression (LR), decision trees (DT), multilayer perceptron (MLP), linear and quadratic discriminant analysis (LDA, QDA), gradient boosting (GB), XGBoost, *k*-nearest neighbors (*k*-NN), random forests (RF), and Gaussian Naïve Bayes (NBayes). These models are well established for binary classification of vibrational spectra.^47,48^ Because ATR-FTIR spectra acquired at a single sampling point are one-dimensional signals indexed by wavenumber, the CNN was implemented as a 1-D residual network operating along the wavenumber axis, comprising a strided convolutional stem (kernel width 7 points, 28 cm^−1^ at the 4 cm^−1^ resolution used here) and two residual blocks (kernel width 5 points, 20 cm^−1^) followed by global average pooling, dropout, and a single sigmoid output, with 10 633 trainable parameters in total. As this parameter count exceeds the number of independent samples by more than two orders of magnitude, the CNN results are reported as exploratory only.

### 2.6 Training pipeline

Modelling was performed at the patient level, with one spectrum per patient, giving 30 independent samples (10 progressors who developed dysplastic lesions during follow-up and 20 non-progressors). This ensures that replicate measurements from a single individual cannot appear in both training and test partitions. The windowed spectral regions 900–1800 cm^−1^ and 2800–3000 cm^−1^ were used as input (623 variables), which were projected onto a small number of partial least squares (PLS) latent variables computed against the progressor label; PLS is a supervised method and was fitted within training folds only, with the number of latent variables selected by inner cross-validation. A Spearman-correlation pre-filter applied before PLS was additionally evaluated but was found to reduce performance (see Results) and was omitted from the final pipeline. For imbalanced learning, class weights were applied to classifiers that support them (SVM, LR, RF, DT); for the remainder, the synthetic minority oversampling technique (SMOTE) was applied *after* the train/test split, to the training partition only, so that no synthetic sample was derived from a held-out patient. Hyperparameters were tuned by grid search, with balanced accuracy as the selection objective.

### 2.7 Evaluation metrics

Progressor status was taken as the positive class, with a fixed decision threshold of 0.5. Given the class imbalance, balanced accuracy and the Matthews correlation coefficient (MCC) are reported alongside accuracy, sensitivity (recall), specificity, area under the ROC curve (AUC), and F1-score. Model performance was estimated by nested cross-validation: hyperparameters were tuned by four-fold cross-validation within each outer training set, and performance was measured on the held-out outer fold. To characterise the variability introduced by the small sample size, the outer loop was a stratified five-fold cross-validation repeated 20 times (100 outer folds in total), stratified on the four-way category (Hy, Hn, Ly, Ln) so that both baseline risk level and progression status were balanced across folds; we report the mean and 95% percentile interval of each metric. Leave-one-out cross-validation was additionally used to obtain a pooled set of out-of-fold predictions across all 30 patients. To test whether performance exceeded chance, a label-permutation test (1000 permutations, with feature selection re-run inside each) was performed, and pairwise model comparisons used the corrected resampled *t*-test of Nadeau and Bengio, which accounts for the dependence between overlapping training folds.

## 3 Results and discussion

### 3.1 Mean spectra analysis

**Fig. 1:**
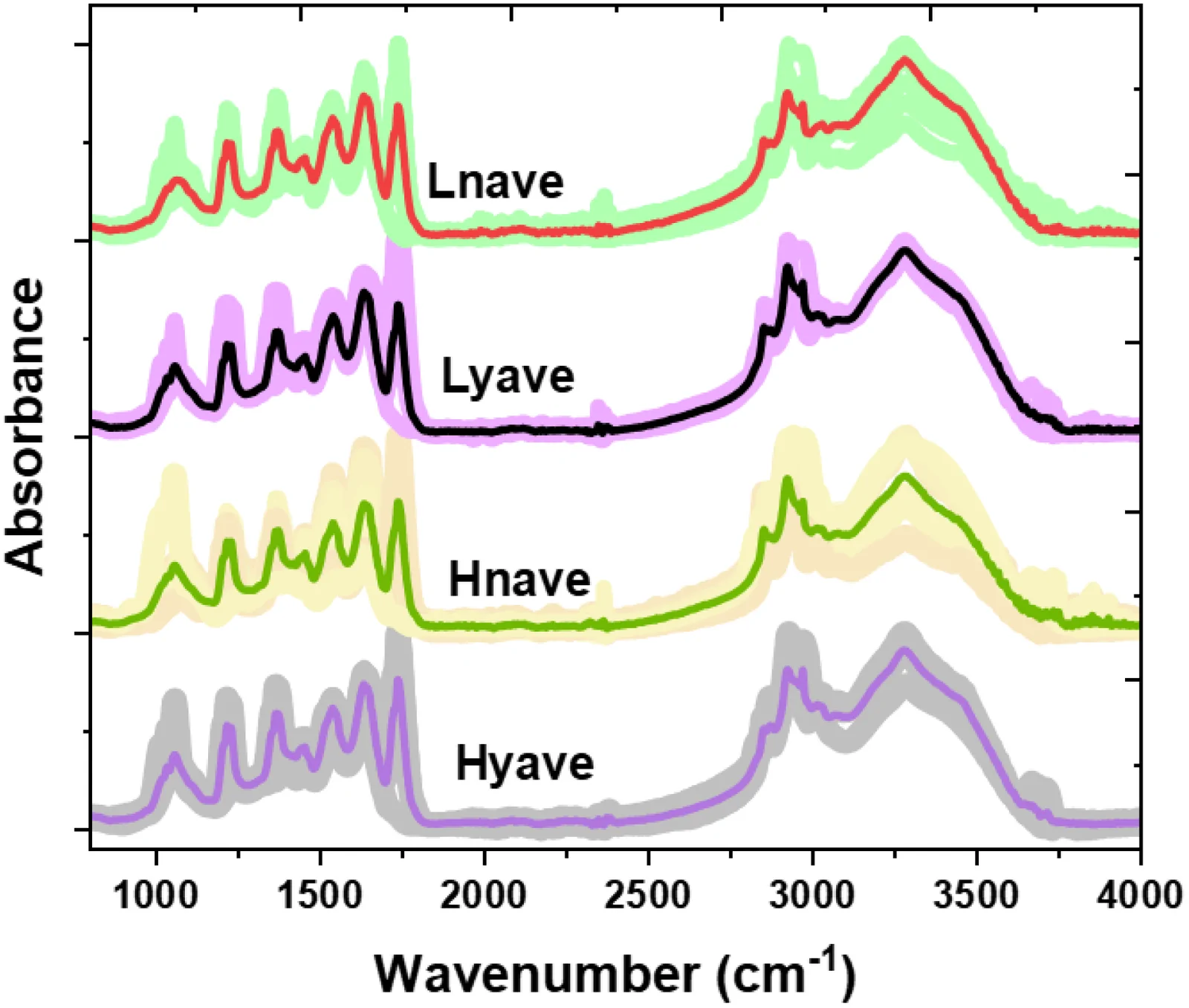
Comparison of MIR absorbance spectra across Hy, Hn, Ly, and Ln groups.

**Fig. 2:**
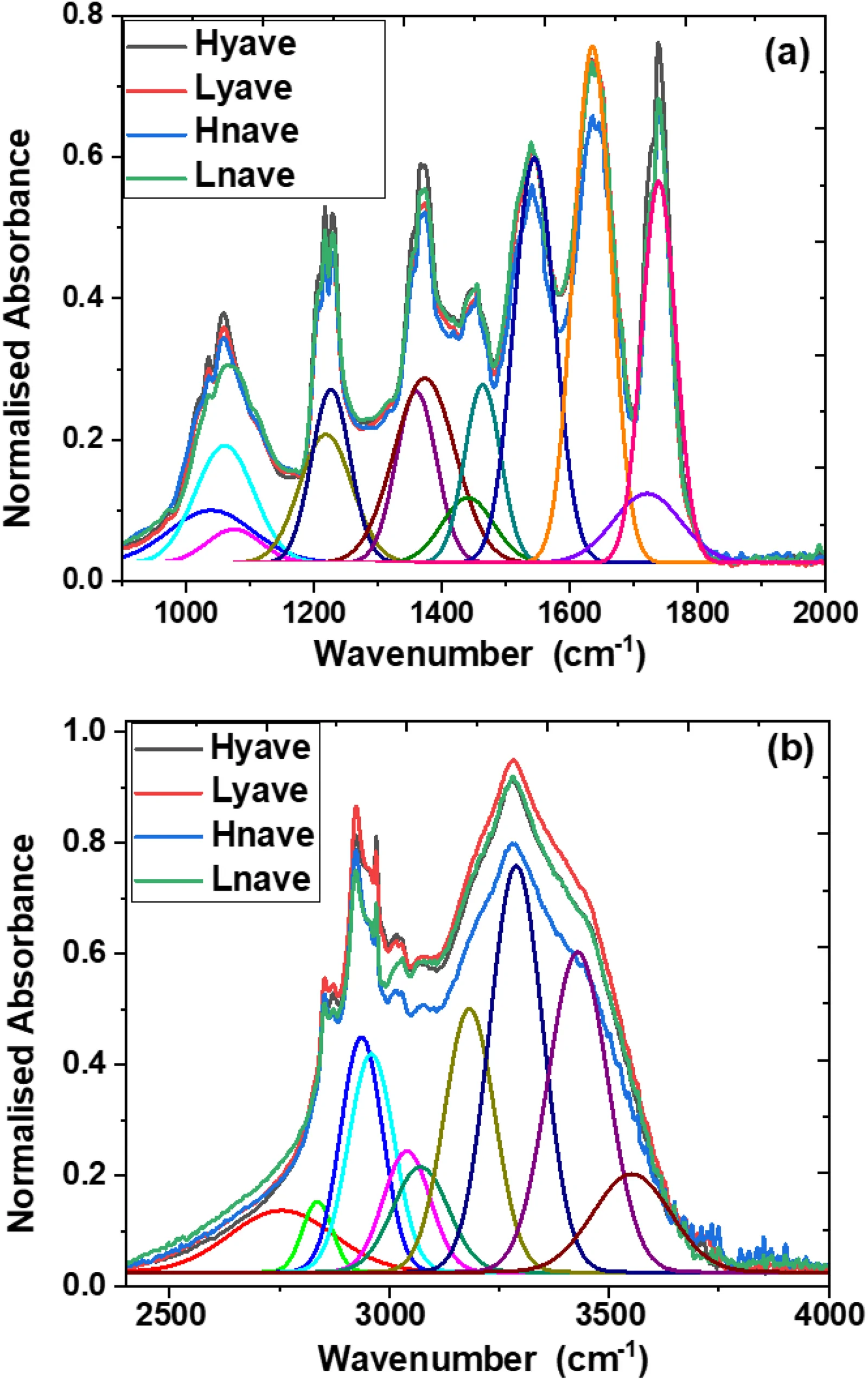
Average FTIR spectra of colonic tissue with Gaussian fitting. Average FTIR spectra of healthy and cancer patients within Hy, Ly, Hn, and Ln groups, fitted with Gaussian curves over (a) 900–2000 cm^−1^ and (b) 2600–3800 cm^−1^.

Fig. 1 presents the ATR-FTIR spectra demonstrating the differences between progressors (those who developed dysplastic lesions on follow up) and non-progressors (those who remained free of dysplastic lesions on follow up) biopsy categories collected from IBD patients for analysis. These groups included Hy, Hn, Ly and Ln categories based on risk status and dysplasia development. High- or low-risk categories were based on the criteria in the British Society of Gastroenterology guidelines applicable at the time of the colonoscopy.^14^Fig. 2 provides a detailed representation of the mean spectra for each category fit with the Gaussian curve using OriginPro software. This highlights the unique spectral biomolecular vibration characteristics of each colorectal tissue type. The mean spectra show slight differences between the non-progressor (Hn/Ln) and progressor (Hy/Ly) tissues regarding molecular vibration peak positions. Approximately 17 prominent molecular features were identified. Notably, these biomolecular fingerprints are found between 900 cm^−1^ and 2000 cm^−1^ and in the region from 2600 cm^−1^ to 3800 cm^−1^ depicted in Fig. 2a and b. The characteristic molecular vibration bands of the colorectal tissues occur at 971, 1024, 1035, 1060, 1080–1085, 1112, 1220, 1240, 1337–1369, 1455, 1540, 1631, 1650, 1717, 1738, 1750, 2853, 2873, 2925, 2970, 3015, 3064, 3200, 3280, 3452 and 3539 cm^−1^.^28,49^

The biochemical signatures observed within the range of 950 to 1180 cm^−1^ are primarily related to the components of DNA and RNA, including ribose and deoxyribose (C–O stretching vibrations). This range also encompasses C–O stretching vibrations found in glycogen and collagen. Also, the molecular vibration peak in the range of 1050 to 1080 cm^−1^ serves as a significant indicator of the extent of oxidative DNA damage.^50^ This highlights differences in ATR-FTIR absorbance between Hy and Ly compared with Hn and Ln samples, reflecting the impact of oxidative stress on DNA structure. Protein contributions were observed in the 1170–1275 cm^−1^ region (associated with amide III due to stretching PO_2_^−^ asymmetric), 1400–1480 cm^−1^ (assigned to CH_2_/CH_3_ deformation vibrations due to lipid),^51^ 1540 cm^−1^ (ascribed to amide II absorption – predominantly β-sheet of amide II), and 1635 cm^−1^ (attributed to β-sheet structure of amide I).^52^ The vibration bands occurring in the range of 2800–3700 cm^−1^ are primarily associated with CH_2_ and CH_3_ stretching vibrations of lipids, including symmetric and asymmetric modes, as well as amide A/B N–H and C–H stretching.^53–56^

**Fig. 3:**
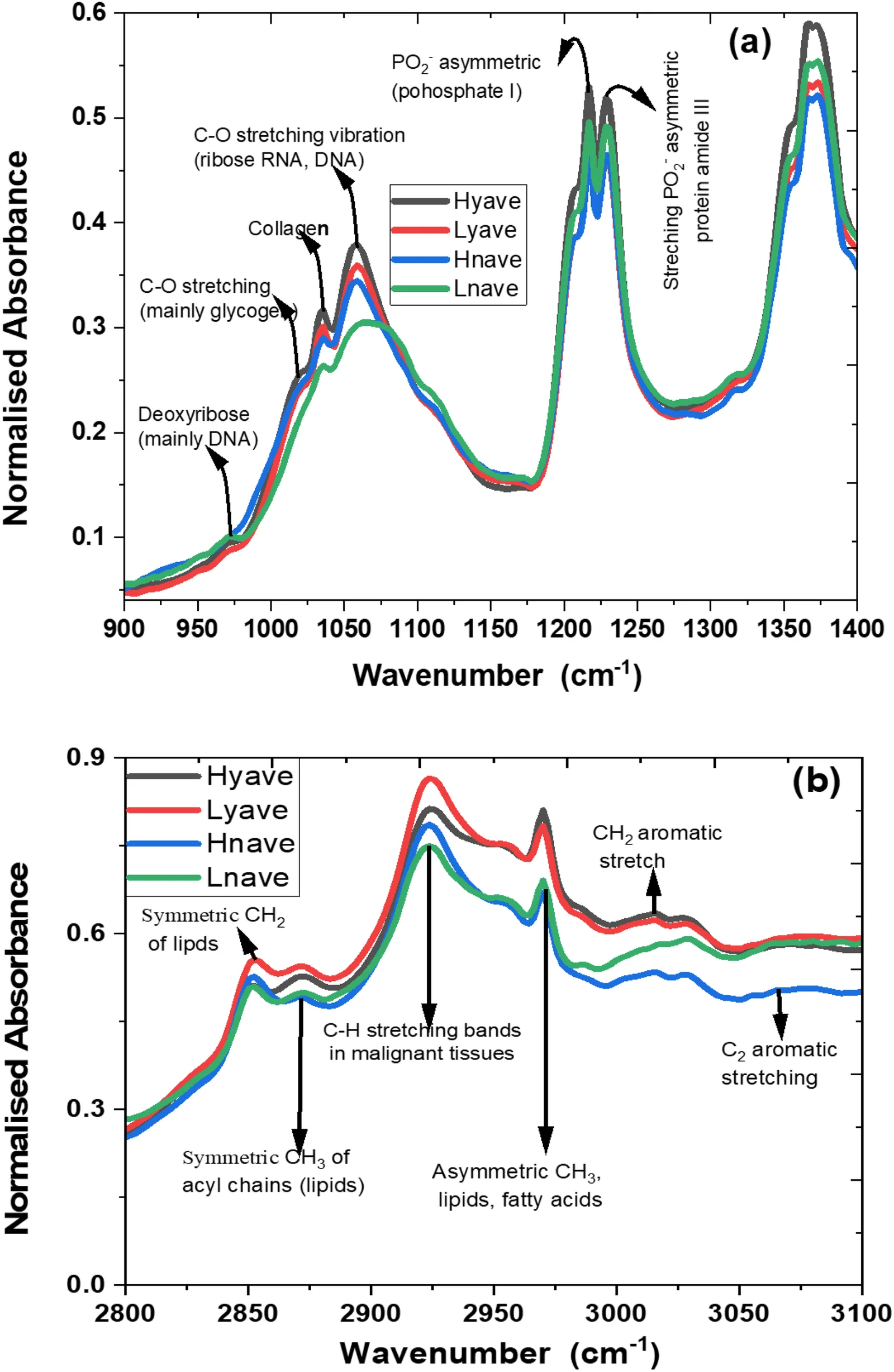
Mean FTIR spectral fingerprint for the most sensitive regions: (a) 900–1200 cm^−1^ and (b) 2800–3100 cm^−1^.

Fig. 3(a) and (b) illustrate the spectral range where IBD patients who developed precancerous lesions (Hy and Ly) showed higher relative intensity from patients who did not develop any precancerous lesions on follow-up (Hn and Ln). The biomolecular vibration bands at elevated levels in the rectal biopsies of progressors (Hy and Ly) occurred at 1034 cm^−1^ (collagen), 1060 cm^−1^ (symmetric and asymmetric stretching of PO_2_^−^ phosphodiester backbones in DNA and RNA), 2874 cm^−1^ (stretching C–H, N–H and symmetric stretching vibration of CH_2_ acyl chains (lipids)), 2924 cm^−1^ (symmetric stretching vibration of CH_3_ and C–H stretching bonds in rectal biopsies of progressors (Hy and Ly)), 2970 cm^−1^ (asymmetric vibration of CH_3_ in lipids and fatty acids) and 3015 cm^−1^ (CH vibration of lipids) compared to rectal biopsies of non-progressors (Hy and Ly).^28,29,57^ These molecular fingerprints are summarised in Table 2. The polysaccharide-cellulose peak at 1060 cm^−1^ in the IBD patients who progressed to develop precancerous lesions was more pronounced. Similarly, the changes in the relative intensity of the absorbance spectrum at 2924 cm^−1^ were observed in the C–H stretching bands in the rectal biopsies of progressors (those who developed precancerous lesions on follow-up). This increase in FTIR absorbance in the Hy and Ly groups is attributed to changes in colonic protein, lipid and fatty acid composition. The variation in the absorbance intensity values of specific structures shown in Fig. 3 agrees with Lasalvia *et al.*^58^ The authors reported similar increases in nucleic acids, collagen, protein and lipid vibration peaks in malignant tissue compared to those in healthy IBD patient tissues.

**Table 2:** Detailed biomolecular fingerprints illustrating distinct vibrational peaks associated with key biomolecules, including lipids, proteins and DNA. These spectral features reveal differences between high- and low-risk patients, highlighting potential biological variations associated with high-risk colorectal cancer (CRC)

Wavenumber (cm^−1^)	Assignment	Vibration type	Source
971	*ν*PO_4_	Nucleic acids/phosphorylated proteins	59
1035	*ν*(CC), *ν*(CH_2_OH)	Skeletal stretching, polysaccharides	60
1060	C–O stretch	DNA/RNA (deoxyribose)	60
1076	*ν*PO_2_^−^ (sym)	Phosphodiester groups in nucleic acids	59, 61 and 62
1080–1085	C–O–C stretch	Nucleic acids, phospholipids	61
1112	C–OH stretch	Oligosaccharides	63
1220	*ν*_as_PO_2_^−^	Asymmetric phosphate vibration	60
1230	*ν*_as_PO_2_^−^	DNA/RNA phosphate, amide III overlap	62
1367–1369	CH_2_ bending	Proteins	64
1455	C–O–H	Amino acid side chains	64
1540	Amide II	N–H bending, C–N stretching	64 and 65
1631	Amide I	β-Sheet structure	64
1646–1650	Amide I	Proteins, nucleic acids	59
1717	Amide I	C=O stretching	64
1738	*ν*(C=O)	Lipids, esters	62
1750	*ν*(C=C)	Lipids, fatty acids	66
2853	*ν*_s_CH_3_	Lipids, biomolecules	66
2873	*ν*_s_CH_3_	Fatty acids	66
2925	*ν*_as_CH_3_	DNA–lipid interactions	62 and 66
2954	CH_3_ (asym)	Lipids	66
2970	CH_3_ (asym)	Fatty acids	66
2987	CH_3_ (asym)	Lipids, proteins	66
3015	=C–H stretch	Unsaturated lipids	62
3029	O–H/N–H	Water, proteins	64
3064	Amide B	Protein N–H stretching, C_2_ aromatic stretching	65
3200–3500	H_2_O	Symmetric and asymmetric vibrations attributed to water	28
3280	O–H (sym)	Stretching O–H symmetric	28
3452	O–H (asym)	Stretching O–H asymmetric	28
3539	O–H (asym)	Stretching O–H asymmetric	65

**Fig. 4:**
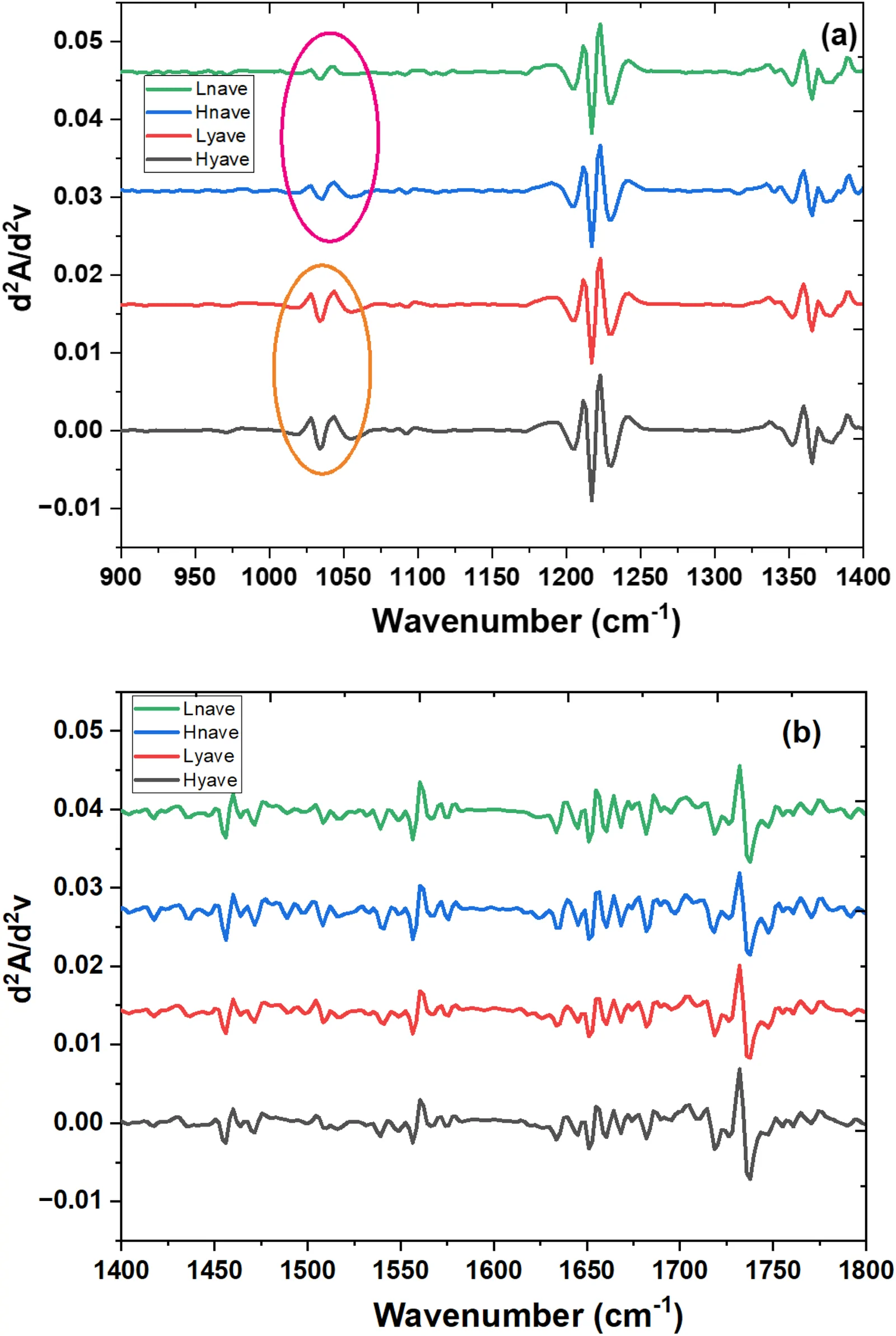
Average of second derivative FTIR spectra highlighting major wavenumbers. Average second derivative FTIR spectra showing major wavenumbers for (a) 1000–1400 cm^−1^ and (b) 1650–1770 cm^−1^.

The second derivative of the absorbance spectra of the four patient groups was performed to identify specific spectral features of small and closely spaced absorption peaks, which are not resolved in the original spectrum. Fig. 4 illustrates the averages of each group's second-derivative spectra for IBD patients. The prevalent wavenumbers detected in the Hy, Ly, Hn and Ln patients were found at 1018, 1033, 1205, 1217, 1230, 1358, 1367, 1380, 1657, 1660, 1670, 1684, 1685, 1717, 1736 and 1745 cm^−1^ as seen in Fig. 4a and b. The change in magnitude of the peaks at 1033 cm^−1^ and 1054 cm^−1^ is of higher relative intensity in the Hy and Ly patient (malignant) groups compared to the Hn and Ln (normal) groups, as shown in Fig. 4(a). These peaks are assigned to *ν*(CO) skeletal *cis* conformation, *ν*(CH_3_OH), and *ν*(CO) stretching coupled with C–O bending.^67,68^ They are also associated with PO_2_^−^ stretching vibrations.^69,70^ The magnitude of molecular vibrations at specific wavenumbers (1217, 1230, 1367, and 1738 cm^−1^) increased in sample Hy, while the rest remained relatively the same in other samples. These peaks are ascribed to PO_2_^−^ asymmetric (phosphate I) vibrations arising from DNA, including asymmetric PO_2_^−^ stretching (phosphate I), C–O stretching, C–H deformation, N–H deformation, and C=O stretching (lipids) damage. The remaining bands in the second-derivative spectra depicted in Fig. 4(b) showed minimal intensity variation, indicating a consistent spectral pattern across groups.

### 3.2 Area under molecular vibration and peak intensity ratios analysis

The integrated peak areas beneath the biochemical vibrational peaks of the FTIR-ATR spectrum were analysed for the four classification categories. This analysis allowed for the conversion of raw spectral data into biologically significant spectral biosignatures. These spectral biosignatures effectively quantified to determine the alterations in tissue composition, providing insight into the biochemical changes occurring within the samples. Therefore, the proportional ratio of two ATR-FTIR integrated peak areas (*P*_a_ and *P*_b_) was determined as follows:1
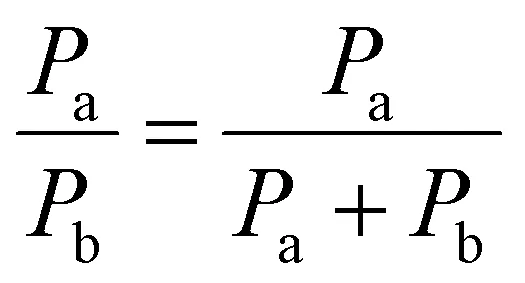


The following integrated absorbance peak area proportions ratios were calculated: P1060/P1230, P1060/P1540, P1455/P1540, P1633/P1217, P1633/P1540, P2959/P1540 and P2926/P2970, as depicted in Table 3. The aim of using the proportional ratio is to determine the fractional contribution of a specific functional group relative to the combined total of the two vibration bands. The proportional ratios of P1455/P1540, P1633/P1217, P1633/P1540, and P2959/P1540 were slightly higher in the Hy and Ly colorectal tissues than in the Hn and Ln samples. This suggests that the relative proportions of the amino acid chains at 1455 cm^−1^, the amide I at 1633 cm^−1^, and the asymmetric CH_3_ stretching vibration of lipids, DNA and proteins (2959 cm^−1^) are elevated in patients at high risk of colorectal cancer compared to Hy and Hn patients. Similarly, the proportional ratio of β-sheet structure of amide I (1633 cm^−1^) to asymmetric phosphate I (PO_2_^−^) band (1540 cm^−1^) showed a slight increase in IBD patients who subsequently developed precancerous lesions. In contrast, the P1060/P1540 absorbance proportional ratio exhibited no significant change between Hy and Hn groups but decreased in IBD patients within the Ln group compared to the Ly group. Furthermore, the relative content ratios of the C–O stretch DNA/RNA (deoxyribose) (1060 cm^−1^) to DNA/RNA phosphate, amide III overlap (1230 cm^−1^) were significantly higher in both Hn_ave_ and Ln_ave_ samples than in Hy_ave_ and Ly_ave_ samples. By employing proportional ratios, variations in the amounts of lipids, proteins, nucleic acids, and carbohydrates caused by cancer can be used more reliably to differentiate healthy, benign, and malignant tissues than relying solely on absolute peak intensities.

**Table 3:** Area under the dominant molecular vibration band ratios

Peak ratios	Hy	Hn	Ly	Ln
P1060/P1230	0.38	0.43	0.35	0.43
P1060/P1540	0.38	0.38	0.36	0.33
P1455/P1540	0.42	0.44	0.43	0.60
P1631/P1217	0.58	0.59	0.61	0.59
P1631/P1540	0.57	0.54	0.56	0.54
P2954/R1540	0.57	0.39	0.58	0.55
P2925/P2970	0.50	0.46	0.52	0.48

**Fig. 5:**
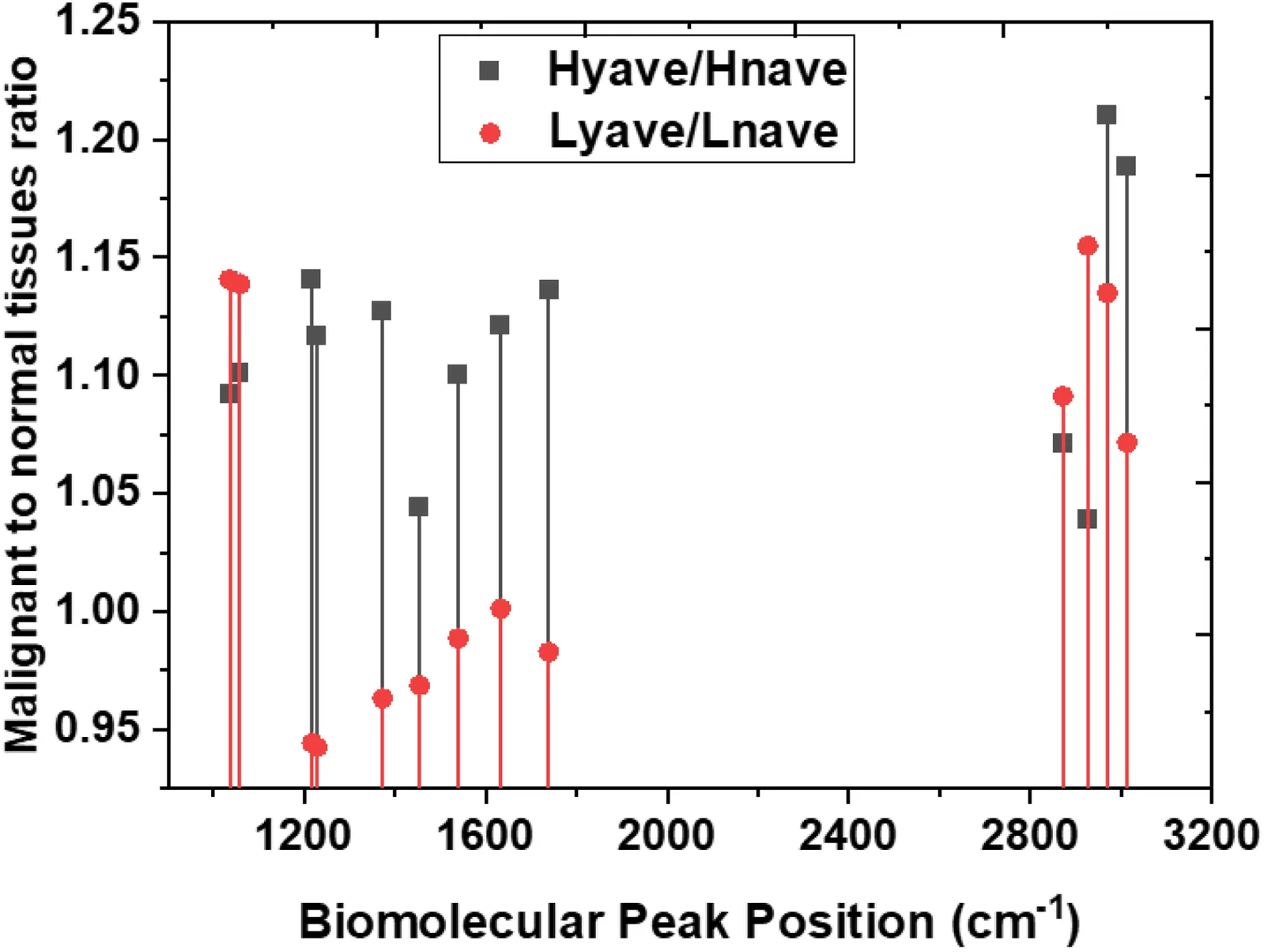
Biomolecular vibration band peak intensity ratio in IBD-associated CRC. Biomolecular vibration band peak intensity ratio comparing IBD-associated CRC patients to healthy controls.

The molecular vibration absorbance of the most pronounced peak ratios of the Hy_ave_/Hn_ave_ and Ly_ave_/Ln_ave_ was determined for peaks centered at 1035, 1058, 1218, 1228, 1373, 1454, 1540, 1633, 1740, 2872, 2926, 2970 and 3014 cm^−1^. Fig. 5 illustrates a pair comparison plot of the Hy_ave_/Hn_ave_ and Ly_ave_/Ln_ave_ against various dominant absorbance peaks in Fig. 2. The molecular vibration bands ratio ranges between 1200 cm^−1^ and 1750 cm^−1^ and 2970 cm^−1^ and 3200 cm^−1^ were significantly higher in the Hy_ave_/Hn_ave_ than in Ly_ave_/Ln_ave_. The 1200 cm^−1^ to 1750 cm^−1^ and 2900 cm^−1^ to 3020 cm^−1^ spectral ranges are associated with pronounced levels of carbohydrates or glycoprotein, asymmetric PO_2_^−^ stretching in RNA, amide III, amide II (protein), amide I (protein), C=O stretching bands (protein), and asymmetrical methyl groups in the high-risk patients who had CRC. The study indicates a relationship between nucleic acid symmetry and the symmetry CH_2_ vibration bands at wavenumbers, specifically 1080 cm^−1^, 1035 cm^−1^, 2872 cm^−1^, and 2926 cm^−1^. It finds that as the Ly_ave_/Ln_ave_ ratio increases, the Hy_ave_/Hn_ave_ ratio at these vibration bands decreases. This suggests an inverse correlation between these ratios, highlighting a noteworthy interaction between nucleic acids’ vibrational characteristics and the symmetry CH_2_ vibration bands.

### 3.3 Multivariate statistical analysis

#### 3.3.1 PCA on the IBD-associated tissues

**Fig. 6:**
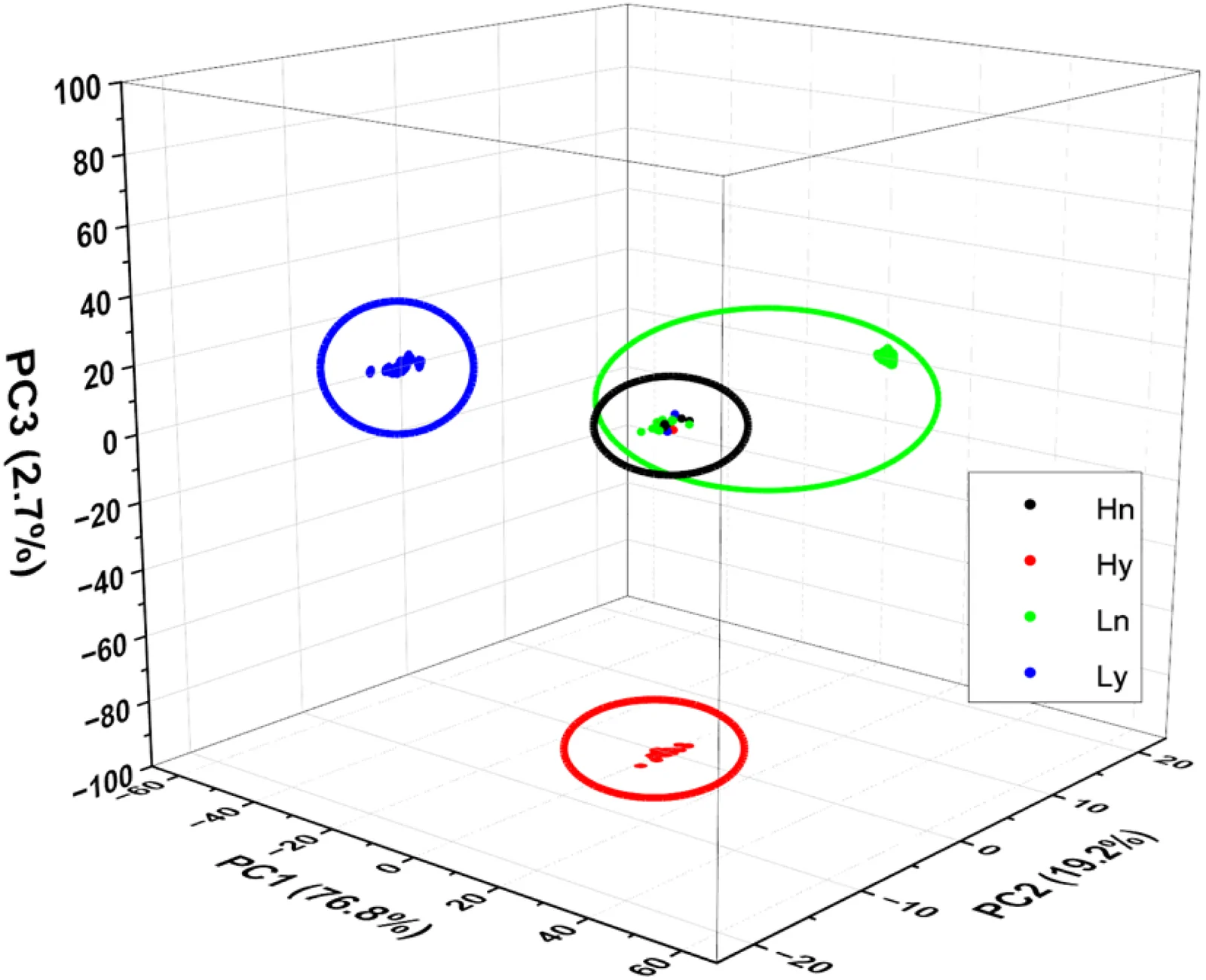
Principal component analysis (PCA) of FTIR-ATR spectra. PCA of FTIR-ATR spectra for different patient classifications across the wavenumber range 900–4000 cm^−1^, showing factorial coordinates PC1, PC2, and PC3.

The multivariate statistical method, such as PCA, extracts valuable information from large FTIR datasets. By transforming complex FTIR data into simpler, manageable components, PCA helps to illuminate patterns and relationships within the data that might otherwise remain hidden. Fig. 6 represents the three-dimensional PCA scatterplot analysis of Fig. 2, with the peak assignments shown in Table 1. It exhibits a distinctive separation between the different groups of colonic IBD patients. The proportion of variance accounted for by the first three principal components, PC1, PC2 and PC3, is 76.8%, 19.2% and 2.7%, respectively. The contribution from the stretching vibrations of amide I (1520–1698 cm^−1^) and CH_3_/O–H (2800–3400 cm^−1^) bending of methyl and water groups dominates PC1. Amide and water may not be potential contributors to PC1; nonetheless, the nucleic acid (PO_2_^−^), occurring between 900–1150 cm^−1^ and centered at 1060 and 1080 cm^−1^, may also be present in PC1. PC1 accounted for 76.8% of the total variance. PC2 is prevalent with nuclei and protein contributions occurring at 971 cm^−1^, 1045 cm^−1^, and 1056 cm^−1^, as well as proteins at 1230 cm^−1^ – amide III (α-helix), and 1540 cm^−1^ – amide II as depicted in Fig. 3. PC3 encapsulates a wealth of biological information derived from various sources, primarily nucleic acids and protein vibrations. This intricate interplay of molecular components contributes significantly to our understanding of biological systems, accounting for 2.7% of the overall variance observed in the IBD-associated colorectal tissue data. The C–O–C stretching (nucleic acids and phospholipids) and PO_2_^−^ asymmetric stretching vibration bands peaked at 1080–1085 cm^−1^ and 1238 cm^−1^, showing a strong influence of variation. This can be ascribed to oxidative-induced damage to DNA and RNA.^71–74^

The results of the PCA analysis indicate a clear distinction between the patient groups designated as Hy and Ly, compared to those classified as Hn and Ln, which are clustered. This separation highlights the unique characteristics and behaviors of the Hy and Ly groups, further emphasising their differences from the Hn and Ln cohorts. In this study, we illustrate how three distinct principal component analyses may be effectively used to differentiate between patients with IBD who are at a heightened risk of developing CRC. This approach enhances our understanding of the risk factors associated with CRC in IBD patients. It is a valuable tool for identifying individuals who may benefit from closer monitoring and preventive interventions.

#### 3.3.2 Limitation related to variability within the groups

A limitation of this study is the higher variability observed in the Ln group compared to other groups. This greater dispersion may reflect biological differences, sample variation, or other uncontrolled factors not fully captured in this dataset. While the main trends and conclusions are consistent, the increased variability could have reduced statistical power and led to wider confidence intervals for some comparisons. As a result, view the findings for the Ln group with caution. Future research with larger, more uniform samples would help confirm these results and reduce uncertainty.

#### 3.3.3 HCA on the IBD-associated tissues

**Fig. 7:**
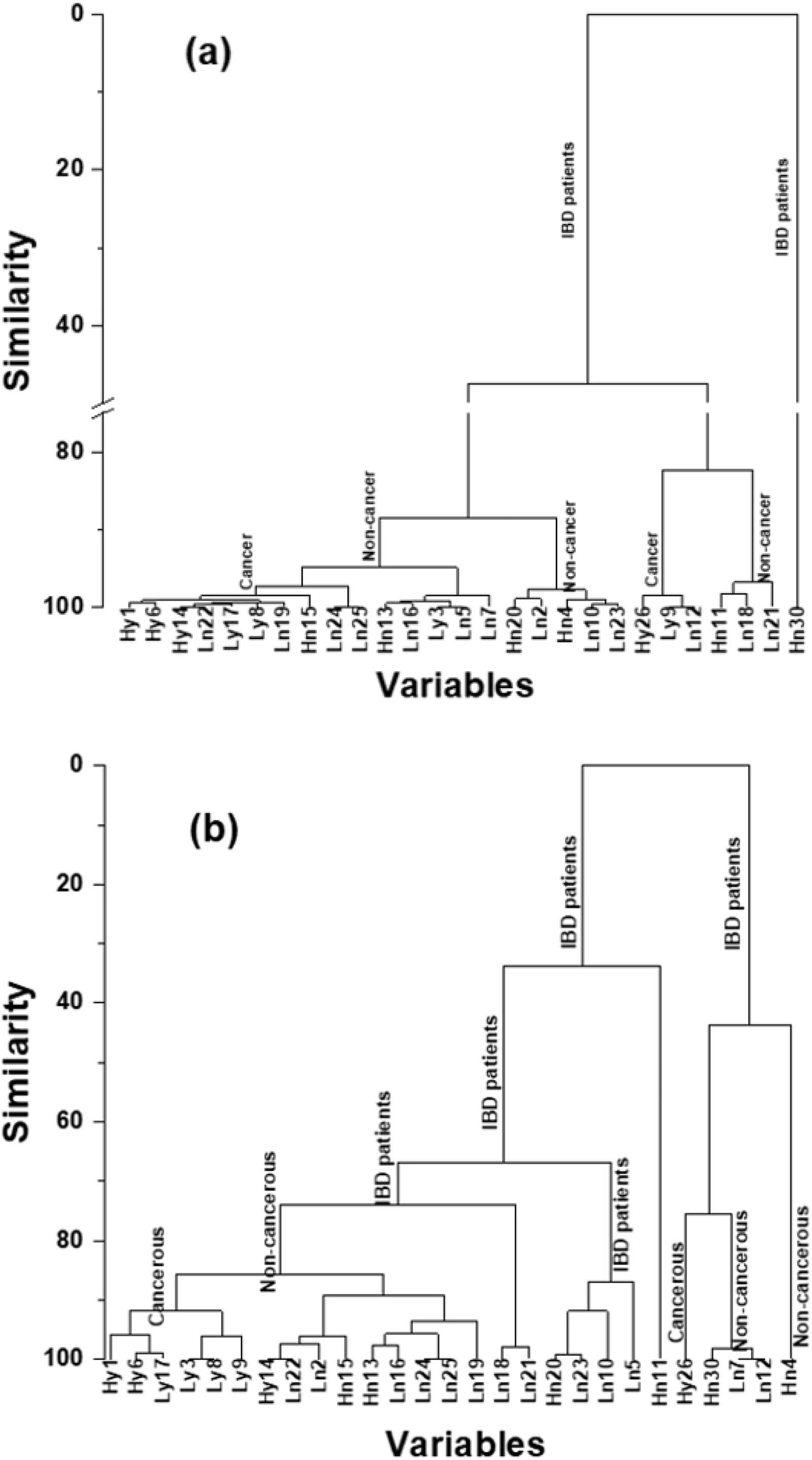
Dendrographic classification of ATR-FT-IR spectra. Dendrograms of ATR-FT-IR spectra for healthy and malignant IBD-related CRC biopsy tissue samples over the wavenumber ranges (a) 900–2000 cm^−1^ and (b) 2500–3200 cm^−1^.

The HCA clustering method identifies groups of similar sample data, organises them into clusters, and depicts a hierarchy among them (Yang, 2021).^32^ HCA results are typically presented as dendrograms, which organise samples hierarchically based on spectral similarity. The differences in peak intensities and wavenumber shifts of biomolecules from the FTIR spectral data highlight the variance between malignant groups (Hy and Ly) and their control counterparts (Hn and Ln). HCA visually represents spectral relationships at both group and subgroup levels. The FTIR spectra of colorectal tissues from high-risk and low-risk IBD patients, shown in Fig. 1, were vector normalised. This was followed by applying Ward's algorithm as an amalgamation rule and Euclidean distances for clustering and creating dendrograms.^75^Fig. 7(a) shows HCA dendrographic results obtained from different IBD patient groups within the 900 to 2000 cm^−1^ spectral range. Using this approach, only two groups of Hy/Ly (precancerous) and Hn/Ln (not precancerous) are formed, with the Hn/Ln categories further divided into subgroups. Nevertheless, two cancerous samples (Ly3 and Ly6) fall within the non-cancerous subgroup. Similarly, Fig. 7(b) illustrates the dendrogram for HCA using FTIR spectral ranging from 2500–3200 cm^−1^. The HCA dendrogram displayed the intragroup similarity found among IBD patients with cancer (Hy/Ly), and non-cancerous (Hn/Ln) samples organised into clusters. These results are similar to the PCA results, where the Hn and Ln samples are classified into one group. Meanwhile, the HCA dendrogram indicates clear differences among the different groups of the investigated IBD patient colorectal tissue samples across the two spectral ranges.

### 3.4 Binary classification and statistical significance

Table 4 summarizes performance across the different models under repeated nested cross-validation, reported as the mean and 95% percentile interval over 100 outer folds. Balanced accuracy ranged from 0.61 to 0.70, with LDA nominally highest (0.70) and the tree ensembles lowest (RF and GB, 0.61). The intervals were wide (typically spanning ∼0.25 to 1.00) and overlapped substantially between all models, and the MCC intervals extended below zero for every classifier, reflecting the constraint imposed by a held-out partition of six patients.

**Table 4:** Classification performance under repeated nested cross-validation, reported as mean [95% percentile interval] over 100 outer folds. Progressor is the positive class

Model	Accuracy	Balanced accuracy	Sensitivity	Specificity	AUC
SVC	0.69 [0.33, 1.00]	0.68 [0.31, 1.00]	0.64 [0.00, 1.00]	0.71 [0.25, 1.00]	0.73 [0.12, 1.00]
LR	0.67 [0.33, 1.00]	0.66 [0.38, 1.00]	0.63 [0.00, 1.00]	0.69 [0.25, 1.00]	0.73 [0.38, 1.00]
MLP	0.69 [0.33, 1.00]	0.66 [0.25, 1.00]	0.57 [0.00, 1.00]	0.74 [0.25, 1.00]	0.75 [0.25, 1.00]
DT	0.65 [0.33, 1.00]	0.61 [0.25, 1.00]	0.50 [0.00, 1.00]	0.72 [0.25, 1.00]	0.63 [0.12, 1.00]
RF	0.66 [0.33, 1.00]	0.61 [0.25, 1.00]	0.45 [0.00, 1.00]	0.76 [0.37, 1.00]	0.68 [0.06, 1.00]
GBoost	0.66 [0.33, 0.83]	0.61 [0.25, 0.88]	0.48 [0.00, 1.00]	0.74 [0.25, 1.00]	0.71 [0.15, 1.00]
KNN	0.65 [0.33, 1.00]	0.65 [0.25, 1.00]	0.66 [0.00, 1.00]	0.64 [0.25, 1.00]	0.68 [0.25, 1.00]
LDA	0.69 [0.33, 1.00]	0.70 [0.31, 1.00]	0.73 [0.00, 1.00]	0.66 [0.25, 1.00]	0.78 [0.43, 1.00]
QDA	0.66 [0.33, 1.00]	0.62 [0.25, 1.00]	0.49 [0.00, 1.00]	0.74 [0.25, 1.00]	0.72 [0.25, 1.00]
NBayes	0.67 [0.33, 1.00]	0.65 [0.25, 1.00]	0.60 [0.00, 1.00]	0.70 [0.25, 1.00]	0.71 [0.31, 1.00]
XGBoost	0.67 [0.33, 1.00]	0.65 [0.25, 1.00]	0.56 [0.00, 1.00]	0.73 [0.25, 1.00]	0.74 [0.18, 1.00]
1-D CNN	0.53 [0.17, 0.83]	0.46 [0.12, 0.75]	0.51 [0.00, 1.00]	0.41 [0.00, 1.00]	0.54 [0.15, 0.93]

Crucially, a label-permutation test showed that this performance was not distinguishable from chance. For XGBoost, the observed balanced accuracy fell close to the mean of the permutation null distribution, giving *p* = 0.51; that is, once feature selection is re-run inside each permutation, a pipeline of this flexibility reproduces the observed performance on randomly-labelled data approximately half the time. Consistent with this, pairwise comparisons using the corrected resampled *t*-test revealed no statistically significant differences between classifiers after Bonferroni correction (smallest adjusted *p* = 0.38). We further note that the exact McNemar test, applied to a held-out partition of this size, cannot in principle reach conventional significance: for the numbers of discordant pairs observed here, the smallest attainable exact *p*-value did not fall below 0.05, so model-to-model comparisons on a single split are uninformative and all comparative statements are based on the repeated-resampling intervals instead.

**Fig. 8:**
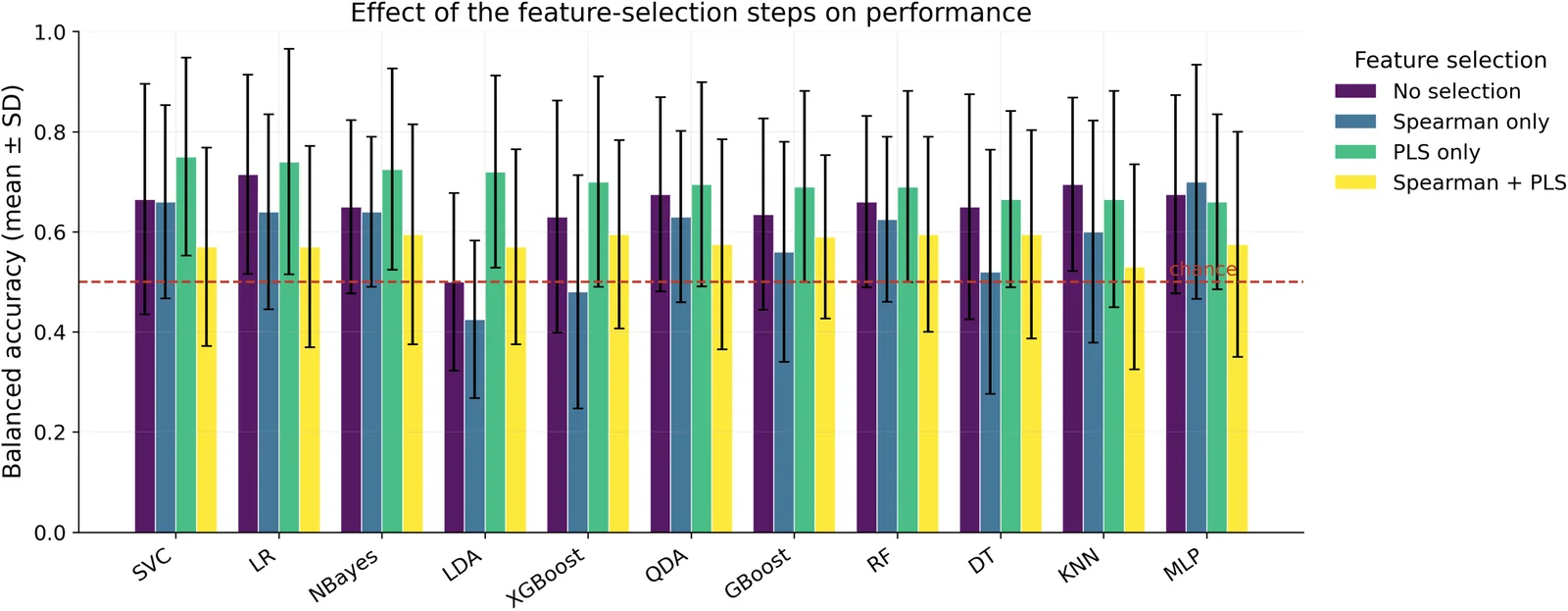
Feature-selection ablation. Mean balanced accuracy per model under four input conditions: no selection, Spearman pre-filter only, PLS only, and Spearman followed by PLS. PLS alone is best; the Spearman pre-filter is detrimental.

The feature-selection ablation provided a clear methodological result (Fig. 8). PLS projection alone gave the highest balanced accuracy for almost every classifier (*e.g.* SVM 0.75, LR 0.74, NBayes 0.73, LDA 0.72), whereas adding the Spearman pre-filter consistently reduced performance (0.53–0.60 across models); using the full windowed spectrum without dimensionality reduction was intermediate. We therefore retained PLS projection alone in the final pipeline. Feature-importance analysis showed strong agreement between two supervised routes—PLS variable importance in projection (VIP) and the classifiers’ own back-projected importances (Spearman rank correlation 0.97)—both identifying the 2950–3030 cm^−1^ region (CH_2_/CH_3_ and =C–H stretching of lipids and fatty acids) as most discriminative, consistent with the band assignments in Table 2.

The 1-D CNN did not exceed the classical classifiers and performed below chance (balanced accuracy 0.46, MCC −0.09), which is unsurprising given its parameter count relative to the sample size; we regard the deep-learning results as exploratory only. The principal limitation of this study remains the sample size: 30 patients with 10 progressors cannot support a claim of clinical validity, and the results should be interpreted as hypothesis-generating—indicating that ATR-FTIR spectra of rectal biopsies contain lipid-region variation associated with subsequent dysplasia, and quantifying the uncertainty on that association, rather than establishing a deployable classifier. Endoscopic risk scores did not always align with histopathological outcomes, and incorporating richer metadata (detailed patient records, IBD severity, disease duration) within a multimodal framework, together with a larger multi-center cohort, will be required to establish whether this signal supports classification.

## 4 Conclusions

In conclusion, ATR-FTIR spectroscopy was applied to IBD-associated rectal tissue samples to distinguish patients who progressed to develop precancerous (dysplastic) lesions from those who did not over a 6-year follow-up period. This approach demonstrates potential as a complementary diagnostic tool for identifying IBD patients at elevated risk of developing CRC. Analysis of absorbance peak ratios revealed compositional differences in rectal tissues, particularly within the 1200–1700 cm^−1^ and 2900–3100 cm^−1^ regions, indicating variations in nucleic acid, protein, and lipid-related vibrations. Multivariate methods, including principal component analysis (PCA) and hierarchical clustering analysis (HCA), provided meaningful insights into spectral patterns associated with disease progression. These unsupervised techniques facilitated identification of molecular fingerprints linked to increased CRC risk. In addition, supervised machine learning models achieved balanced accuracy in the range 0.61–0.70 under repeated nested cross-validation, but a permutation test showed this performance was not significantly above chance (*p* = 0.51), and no model significantly outperformed another.

Overall, the integration of chemometric analysis and machine learning with ATR-FTIR spectroscopy shows promise for risk stratification in IBD-associated CRC, though the present results are hypothesis-generating rather than evidence of clinical readiness: they suggest that lipid-region spectral features carry information associated with future dysplasia, while demonstrating that a definitive classifier cannot be established from a cohort of this size. Future studies will evaluate the robustness of this approach in larger and more diverse patient cohorts, investigate sources of inter-patient variability, and assess its ability to discriminate between different stages and subtypes of IBD-associated neoplasia. A larger multi-center follow-up study will further determine the generalizability and clinical utility of these findings.

## Author contributions

E. K. B. and R. T. contributed equally to this work. E. K. B. performed spectroscopy, analysis, and drafted the manuscript; R. T. developed machine learning models; S. A. supervised the machine learning models; A. J. and V. S. supervised the study and provided clinical samples.

## Conflicts of interest

There are no conflicts to declare.

## Data Availability

The raw data required to reproduce the above findings are available to be shared upon request. The processed data required to reproduce the above findings are available to download by contacting: e.kumi-barimah@leeds.ac.uk.
